# Biosurfactants Contribute to Distiller’s Corn Oil Recovery during Ethanol Production

**DOI:** 10.1021/acsomega.5c09040

**Published:** 2025-12-12

**Authors:** Vanessa Alves Lima Rocha, Maria Fernanda dos Santos Mota, Rui de Paula Vieira de Castro, Douglas Braga Teixeira, Eduardo de Oliveira Junior, Manuela Moore Cardoso, Daniela de Oliveira Campos, João Monnerat A. R. de Almeida, Denise Maria Guimarães Freire

**Affiliations:** a Laboratório de Biotecnologia Microbiana (LaBiM), 28125 Universidade Federal do Rio de Janeiro, Rua Hélio de Almeida, 40, Cidade Universitária, Rio de Janeiro, RJ 21941614, Brazil; b Laboratório de Intensificação de Processos e Catálise (LIPCAT), 28125 Universidade Federal do Rio de Janeiro, Rua Sydiney Martins Gomes dos Santos, 13, Parque Tecnológico, Cidade Universitária, Rio de Janeiro 21941-859, Brazil; c Chemical Engineering Program, COPPE, Federal University of Rio de Janeiro, Rio de Janeiro, RJ 21941-914, Brazil

## Abstract

Distiller’s corn oil (DCO) is an important coproduct of the ethanol industry. However, this oil is partially emulsified in the fermentation medium and adheres to solid particles during ethanol production. The addition of surfactants can facilitate the DCO removal. This study is the first to investigate the use of biosurfactants, biodegradable molecules with demulsifying properties, for DCO recovery. For this, two rhamnolipid (RML)-type biosurfactants, one mono-RML-rich mixture and the other, di-RML, both produced using DCO as a carbon source, were added into ethanol production at a concentration of 0.5 g/L. The chemical surfactant Tween 80 was also added as a positive control. Both biosurfactants increased the total oil recovery (thin vinasse and solids); moreover, di-RML also enhanced the DCO recovery from thin vinasse as well as Tween 80. Both surfactants did not impact the ethanol productivity. Additionally, di-RML reduced the viscosity of the reaction medium during liquefaction and simultaneous saccharification and fermentation. These findings suggest that di-RML is a viable and eco-friendly alternative to chemical surfactants, promoting enhanced oil recovery and improved process efficiency in the biorefinery and circular economic context.

## Graphical abstract

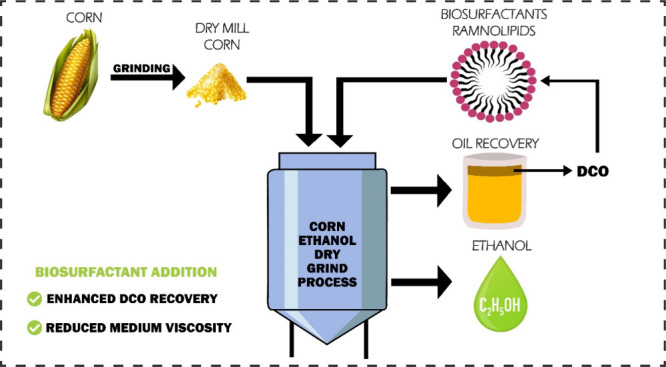

## 1 Introduction

Ethanol is the largest biofuel produced globally, with corn being the most commonly used feedstock. The ethanol process produces whole vinasse as a coproduct in a proportion of around 1:20 L ethanol:whole vinasse.^1^ Usually, the corn ethanol industry subjects this vinasse to centrifugation to separate the coarse solids and the thin vinasse. The coarse solids have part of the yeast solids, corn proteins, corn lipids, and corn fiber, while the thin vinasse has part of the lipids as well as some corn and yeast soluble components.^2^ The coarse solids can be processed to generate dry distiller’s grain (DDG) and/or dry distiller’s grain with solubles (DDGS), which is typically used as animal feed, despite having a higher oil content than is desired for this application. The lipids from the whole or thin vinasse can also be extracted, generating the distiller’s corn oil (DCO). DCO recovery is crucial for enhancing the overall value of the corn ethanol process, presenting application as a feedstock for biodiesel production and animal feed.^2,3^

The most common method for DCO recovery in dry-grind ethanol is the oil separation by centrifugation from the condensed corn distillers’ solubles, a solid obtained after the evaporation of thin vinasse.^4^ However, some drawbacks affect this oil recovery in terms of the yield and quality. During fermentation, DCO appears in various states within the corn slurry, including as oil attached to cell walls and protein matrices as well as oil retained within intact cells, making separation from vinasse challenging. Most of this oil becomes emulsified due to the proteins bound within it, which makes centrifugation insufficient for complete recovery. Emulsifying proteins adhere to the oil droplet surface, forming a robust protective layer that inhibits droplet coalescence. Surfactants improve oil recovery by promoting demulsification through competition with proteins at the emulsion interface, freeing oil droplets.^5–7^ The surfactant ability to promote this demulsification depends on some important surfactant proprieties, such as critical micelle concentration (CMC) and hydrophilic–lipophilic balance (HLB). CMC is the threshold concentration of the surfactant in the solution where micelles start to form; the micelle formation is essential for the destabilization of an existing emulsion and to help the oil extraction to the aqueous phase. HLB is related to the surfactant solubility in water or oil, its influences on the emulsifying capacity, and the type of micelle formation.^8^

Commercial chemical surfactants, such as Tween, are commonly used as DCO recovery agents. Studies have shown that applying Tween 80 (polysorbate 80) to corn slurry results in higher corn oil recovery than conditions without this product.^6^ Chemical surfactants have also been used to recover other vegetable oils; for example, sodium dodecyl sulfate has improved soybean and canola oil recovery.^9,10^ Another study reported surfactants extracting nearly 95% of total oil from ground peanut and canola seeds.^11^ Additionally, Tween 20 has been used to enhance peanut oil recovery.^12^ Commercially, AMS produces demulsifiers like AMulSion COD-B, AMulSion COD-A, and AMulSion COD-G, which are applicable as auxiliaries for corn oil recovery. These surfactants are chemical demulsifiers, some of which are based on hydrophobic silica. Companies such as Evonik also produce oil-in-water emulsifiers, effective as water-in-oil demulsifiers, but these products are highly purified and costly and thus not suitable for the corn ethanol industry.

In the context of sustainable industrial processes, the use of biodegradable compounds is increasingly desirable. Biosurfactants, such as rhamnolipids (RML), are both biodegradable and interfacially active with a molecular structure comprising hydrophilic and hydrophobic components. The hydrophilic headgroup has a strong affinity for polar solvents like water, while the hydrophobic hydrocarbon chain interacts with nonpolar substances like oil. Specifically, RML has one or two (l)-rhamnose molecules as its hydrophilic portion and one or more saturated or unsaturated β-hydroxy fatty acid chains (C_8_–C_24_) as its hydrophobic portion, being called mono-RML or di-RML according to the number of (l)-rhamnose molecules.^13^ These properties allow biosurfactants to exhibit excellent oil recovery and demulsifying capacities. RML is also recognized for its biodegradability, low toxicity, and antimicrobial properties.^14–16^ Furthermore, RML is usually produced from renewable sources, unlike most chemical surfactants.

Previous studies from our research group have shown that DCO can be used as a carbon source by strains of *Pseudomonas aeruginosa* (*P. aeruginosa*) for cell growth, energy maintenance, and rhamnolipid-type biosurfactant (RML) production. DCO was used as the only carbon source for two *P. aeruginosa* strains, one major mono-RML producer, and another major di-RML producer. Both *P. aeruginosa* strains produced RMLs in a concentration of around 5 g/L, and the RMLs presented stability at 80 °C (liquefaction temperature in corn ethanol production) and in a pH range of 4–6 (pH range of the corn ethanol production).^17^

In this context, this study aims to evaluate the effect of two RML-type biosurfactants as additives for ethanol production and its effect on DCO demulsification. This process is in the circular economy context, once it uses a corn ethanol coproduct (DCO) as an additive of the corn ethanol process, aiming to improve this DCO recovery and, consequently, the DDGS quality by decreasing its oil content. This article reports the evaluation of RML-type biosurfactants for DCO removal for the first time.

## 2 Methodologies

### 2.1 Biosurfactants, Raw Material, Reagents, and Standard

The RML-type biosurfactants were produced using two strains of *Pseudomonas aeruginosa*: ATCC 9027 and LFM 1201. The first was obtained from the American Type Culture Collection and primarily produces monorhamnolipids (90% using DCO as a carbon source). The second one is a genetically modified strain from the Institute of Biomedical Sciences, University of São Paulo^18–20^ (National System of Genetic Resource Management and Associated Traditional Knowledge [SisGen] accession record A8D5AF8) and predominantly produces dirhamnolipids (73% using DCO as a carbon source). Throughout this work, the rhamnolipid products from these strains were referred to as mono-RML and di-RML, respectively. The RML production was carried out using DCO as the carbon source in a bioreactor according to the methodology described by Rocha et al.^17^ Both productions generated approximately 5 g/L RML, with a purity of around 10–15% after lyophilization. The RML characterizations were carried out according to the methodology described by Rocha et al.^17^ Mono-RML presented a critical micelle concentration (CMC) of 0.06 g/L and a hydrophilic–lipophilic balance (HLB) of around 7.0, while di-RML presented a CMC of 0.25 g/L and an HLB of around 9.0.

Corn was acquired in a local market. The enzymes α-amylase and glucoamylase (Prodooze, São Paulo, Brazil) were applied in ethanol production. Tween 80 used in this work presented a CMC of 0.2 g/L according to the methodology described by Rocha et al.^17^ Ethanol (Synth, RJ, Brazil) were used as an HPLC standard for the calibration curves. All other reagents were of analytical grade.

### 2.2 Application of Biosurfactants in the Recovered DCO Demulsification

The DCO recovered from an ethanol production process (without surfactant addition) was used for the demulsification test. The DCO demulsification was carried out by adding 2 mL of DCO to a test tube and 2 mL of a biosurfactant solution (mono-RML or di-RML). Different biosurfactant concentrations were tested: in the CMC, 5× CMC, 1 g/L, and 2 g/L. Water was used as a negative control, and the chemical surfactant Tween 80 as a positive control (for this surfactant, the concentration of 1 g/L is equal to 5× CMC). All mixtures were homogenized by vortexing for 1 min to promote the contact of proteins with the biosurfactant and left to rest on a bench at room temperature overnight. The next day, the samples were centrifuged at 5000 rpm for 10 min. Subsequently, the tubes were observed in terms of oil separation, and the precipitated proteins were quantified to compare the demulsification capacities of each condition evaluated. Protein quantification was performed using Bradford’s reagent, and the concentration was evaluated based on a calibration curve.

### 2.3 Ethanol Production with Biosurfactants as an Additive

The following strain of *Saccharomyces cerevisiae* was used for ethanol production: Ethanol Red, from the Genomics and Bioenergy Laboratory (LGE) (Institute of Biology, State University of Campinas–UNICAMP). This microorganism was kept lyophilized at 4 °C until use. The corn was ground in a Willye mill, model TE-680 (Tecnal). The milled corn was sieved in a sieve for granulometric analysis Granutest 420 mm (Telastem). Particles with a particle size of less than 420 mm were used in the experiments.

The ethanol production was carried out in a 1.5 L bioreactor manufactured by New Brunswick Scientific, with a working capacity of 1 L and 300 g/L dry mill corn. The mixture’s pH was adjusted to 5.3. The process commenced with the gelatinization of the corn slurry at 65 °C for 60 min, followed by liquefaction using α-amylase (20 μL/g corn bran) at 80 °C for 90 min. Subsequently, for simultaneous saccharification and fermentation (SSF), the pH of the liquefied pulp was set to 4.9. Glucoamylase (30 μL/g corn bran), urea (0.8 g/L), and yeast (8 mL of activation solution/L) were introduced. Chlorine dioxide (1 mL/L) served as an antibacterial agent. The yeast solution comprised 5 g of dry yeast dissolved in 25 mL of water, undergoing incubation at 32 °C and 150 rpm, for 20 min. Following yeast addition, fermentation of the corn slurry proceeded at 32 °C and 150 rpm for 24 h.

Fermentations in bioreactors containing 0.5 g/L rhamnolipids at the beginning of the process were evaluated. From these experiments, ethanol concentrations were analyzed and compared with the control (without RML). In addition, the effect of the chemical surfactant Tween 80 (polysorbate 80) at concentrations of 0.5 g/L was also evaluated.

The ethanol production yield (Yp/s) was calculated according to eq 1:

$$\mathrm{Yp}/s\;\left(\frac{g}{g}\right)=\frac{g\;\mathrm{of}\;\mathrm{ethanol}}{g\;\mathrm{of}\;\mathrm{dry}\;\mathrm{corn}}$$ 1

The ethanol production volumetric productivity (Qp) was calculated according to eq 2:

$$\mathrm{Qp}\;\left(\frac{g}{L\times h}\right)=\frac{g/L\;\mathrm{of}\;\mathrm{ethanol}}{\mathrm{time}\left(h\right)}$$ 2

### 2.4 Quantification of Ethanol by HPLC

The fermentation samples underwent centrifugation at 10,000 rpm for 10 min, followed by filtration of the supernatants using syringe filters (0.22 μm). Quantitative analysis of ethanol was conducted using high-performance liquid chromatography (HPLC) equipment from Agilent Technologies, series 1200. An HPX-87H column (BioRad, 300 mm × 7.8 mm) was utilized, with the temperature maintained at 65 °C, the flow rate set at 0.6 mL/min, and a detection range from 0 to 500,000 nRIU (refractive index units). The mobile phase consisted of 0.005 M sulfuric acid with a sample injection volume of 20 μL. Calibration curves were employed for the quantification of these analytes.

### 2.5 Corn Oil Recovery by Biosurfactants and Quantification

After fermentation, the ethanol was removed from the samples by evaporation. Afterward, samples were centrifuged at 10,000 rpm for 10 min. The oil from residual solids was extracted by the Soxhlet methodology.^21^

The oil from thin vinasse (obtained from the supernatant) was extracted according to the methodology adapted from Noureddini et al.^22^ For this, to 350 mL of thin vinasse was added 105 mL of hexane. Then, samples were agitated at 180 rpm at 30 °C for 30 min, and the organic phase was separated. After, to 350 mL of thin vinasse was added 105 mL of hexane. Samples were agitated at 180 rpm at 30 °C for 4 h, and the organic phase was separated. Finally, vacuum rotary evaporation was performed to get solvent-free recovered oil for the solid and vinasse methodologies.

For the percentage of total oil extracted, it was considered to be a total oil amount of 3 g in the milled corn used for ethanol production (information provided by the company). Figure 1 illustrates the ethanol production and downstream process.

**Figure 1:**
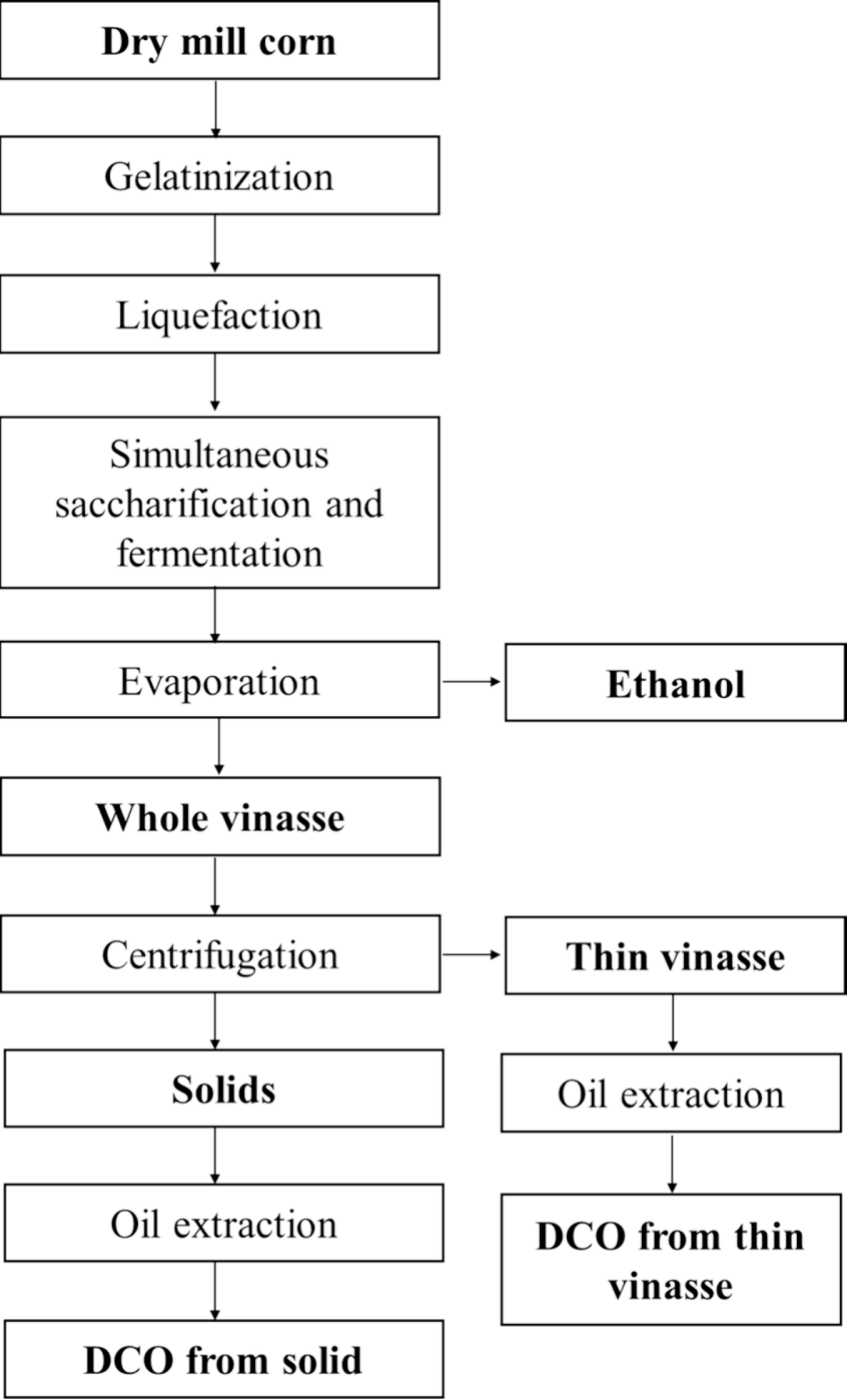
Flowchart of ethanol production, vinasse separation, and oil recovery.

### 2.6 Viscosity Calculation

The viscosity of the corn slurry was measured by taking samples at the end of each ethanol production stage (gelatinization, liquefaction, and simultaneous saccharification and fermentation). Viscosity was measured using a 4 mm orifice Ford cup (Nalgon Equipamentos Científicos, São Paulo, Brasil). Measurements were performed at the temperatures of the ethanol production steps: gelatinization (65 °C), liquefaction (80 °C), and fermentation (32 °C). This analysis was carried out to verify if the addition of biosurfactants reduces the viscosity of the corn slurry.

The calculation of kinematic viscosity (in cSt) was done according to eq 3 (equation provided by the instrument company):

v=3.85×(t−4.49) 3

where *t* is the flow time in s.

### 2.7 Statistical Analysis

Statistical analysis was carried out using Statistica software (v. 7.0) using the Fisher-LSD test, considering a 90% confidence (*p* < 0.10).

## 3 Results and Discussion

### 3.1 Distiller’s Corn Oil (DCO) Demulsification by Biosurfactants

The protein and suspended solid removal capacity by commercial DCO demulsification with the addition of mono- and di-RML surfactants is shown in Figure 2. For comparison, the same procedure was performed with water (negative control) and Tween 80 (positive control). The surfactant demulsification capacity was evaluated in the following concentrations: CMC (0.06 g/L for mono-RML, 0.25 g/L for di-RML, and 0.2 g/L for Tween 80), 5× CMC (0.3 g/L for mono-RML, 1.25 g/L for di-RML, and 1.0 g/L for Tween 80), 1 g/L (16× CMC for mono-RML and 4× CMC for di-RML), and 2 g/L (33× CMC for mono-RML, 8× CMC for di-RML, and 10× CMC for Tween 80).

**Figure 2:**
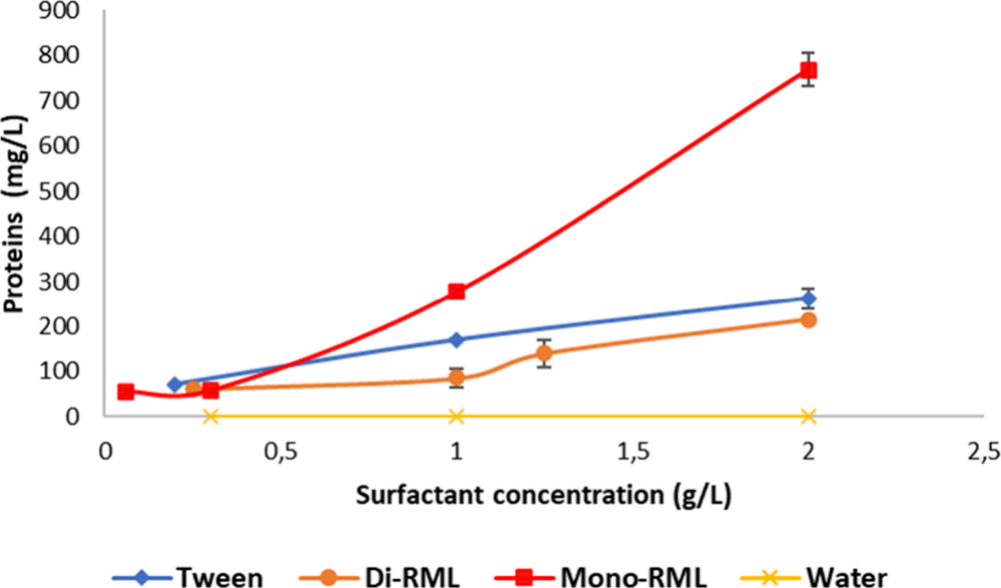
Proteins extracted from distiller’s corn oil (DCO) by the addition of biosurfactants: mono-RML and di-RML. Distilled water was used as a negative control and the commercial chemical surfactant Tween 80 as a positive control.

Mono-RML, di-RML, and Tween 80 were efficient in demulsifying the DCO, precipitating residual proteins that were emulsified in the oil phase. For all the concentrations tested, the protein recovery was significantly higher than the negative control (water), which provided zero protein extraction. The protein extraction increased with the concentration of the surfactant for all surfactants tested. When comparing the surfactants in the CMC, Tween 80 showed statistically the same protein demulsification capacity as di-RML and a greater protein demulsification capacity than mono-RML (*p* < 0.10). However, in the higher concentration evaluated (2 g/L), where mono-RML was in a concentration 33 times higher than CMC, this surfactant extracted around 3 times more protein content than di-RML and Tween 80. This is practically due to the relationship between the CMC and the protein extraction. CMC is the surfactant threshold concentration in solution where micelles start to form spontaneously; further increases in surfactant concentration mostly contribute to micelle formation. Micelle formation is essential for the protein emulsion destabilization and its further extraction from the oil phase.

Removing these proteins and suspended solids improves the quality of the DCO produced, increasing the profitability of the corn ethanol industry and allowing the use of this DCO for other purposes. Furthermore, this extracted protein could be used for other applications such as animal feed supplementation. Moreover, this result suggests that the addition of the biosurfactant in ethanol fermentation could improve the DCO recovery in corn ethanol downstream.

### 3.2 Ethanol Production Using Biosurfactants as an Additive

Ethanol production in bioreactors was carried out with and without a surfactant addition to compare the DCO recovery. For this experiment, the surfactant concentration was fixed at 0.5 g/L to compare the surfactants at the same concentration. This represents a mono-RML concentration around 8 times higher than CMC, a di-RML concentration 2 times higher than CMC, and a Tween 80 2.5 times higher than CMC. Figure 3 shows the total oil recovery as a percentage from ethanol production with and without surfactant addition.

**Figure 3:**
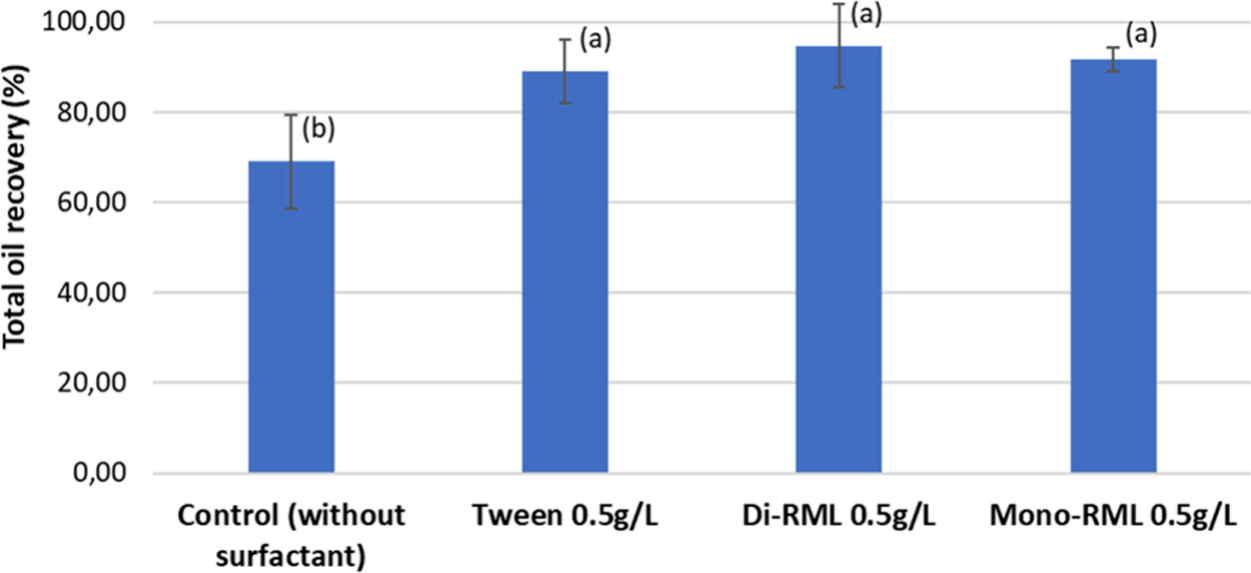
Total oil recovered from whole vinasse (solids and thin vinasse) with application of Tween 80, di-RML, and mono-RML during corn ethanol production. Fermentation without surfactant addition was used as a negative control. Different letters ((a) and (b)) indicate statistically different values (*p* < 0.10).

Significantly higher DCO removal was obtained by adding the surfactant as an additive in ethanol production compared to the condition without a surfactant (*p* < 0.10). In the fermentation without a surfactant, only around 2 g of oil was recovered from the whole vinasse (thin vinasse and solids), representing only around 70% of the total oil expected (3 g). Therefore, over 30% of the oil remained in the residual solid. This quantity of oil retained in the solid is mostly due to the oil retained within intact cells, oil adhered to solid particles, and oil-protein emulsions that hamper the oil extraction. The addition of surfactants enables the recovery of more than 90% of the total oil expected; this higher recovery is probably due to the breakdown of oil-protein emulsions, allowing more oil extraction. Moreover, no statistical difference was observed among the surfactants, confirming that the biosurfactants (mono-RML and di-RML) could be applied in DCO recovery from whole vinasse with the same effectiveness as the chemical surfactant Tween 80.

Table 1 provides a detailed comparison of oil recovery from solid and thin vinasse. Rhamnolipids were particularly efficient in removing oil from solid, with di-RML (56.8%), mono-RML (59.0%), and Tween 80 (53.2%) showing significantly higher recovery rates compared to the control (39.7%).

**Table 1:** Oil Recovery from the Fermented Solid and Thin Vinasse with Application of Tween 80, Di-RML, or Mono-RML during Corn Ethanol Production^a^

condition	oil recovered in solid (%)	oil recovered in thin vinasse (%)
control (without a surfactant)	39.7 ± 10.1^b^	29.4 ± 0.6^b^
Tween 80 0.5 g/L	53.2 ± 3.3^a^	35.9 ± 4.4^a^
di-RML 0.5 g/L	56.8 ± 8.0^a^	37.9 ± 5.6^a^
mono-RML 0.5 g/L	59.0 ± 3.9^a^	32.7 ± 1.5^a,b^

a Results are expressed as means ± SD for triplicates. Different letters in the same column represent significantly different values (*p* < 0.10).

Di-RML and Tween 80 showed higher DCO recovery from thin vinasse compared to the control, which is desirable considering that this fraction is used by most of the dry-grind ethanol plants to recover DCO. Although the addition of mono-RML showed statistically the same recovery of DCO from thin vinasse compared to other surfactants, this value was not statistically significant when compared to the control, even though this surfactant has the lowest CMC among the 3. This different behavior among the surfactants is probably due to HLB, an important property of surfactants closely related to their emulsifying capacity, which expresses the solubility of the biosurfactant in water or oil. HLB ranges from 0 to 20, with molecules with high HLB being more soluble in water, while those with low HLB are more lipophilic.^8^ Therefore, this value predicts the potential applications of a surfactant: values in the range of 4–6 tend to perform better as water-in-oil emulsifiers, 7–9 as wetting agents, 8–18 as oil-in-water emulsifiers, 13–15 as detergents, and 15–18 as solubilizers.^8^ RML molecules have one or more saturated/unsaturated β-hydroxy fatty acid chains (C8–C24) as the hydrophobic part and one or two (l)-rhamnose chains as the hydrophilic part. When the RML has one (l)-rhamnose molecule, it is called mono-RML; when it has two (l)-rhamnose molecules, it is called di-RML. The mono-RML used in this work has 90% of RMLs with only one rhamnosyl group, Rha-C_10_C_10_ being the predominant molecule present, whereas the di-RML used in this work has 73% of RMLs with two rhamnosyl groups, RhaRha-C_10_C_10_ being the primary homologue present. Therefore, di-RML is a more hydrophilic surfactant than mono-RML.^17^ The mono-RML used in this work has an HLB of 7.0, with the di-RML of 9.0 and Tween 80 of 15.^6^ Therefore, the di-RML and Tween 80 are more hydrophilic surfactants than mono-RML, and both tend to form oil-in-water emulsions, where the water is the continuous phase and the oil is the disperse phase. Due to this property, these surfactants probably produce oil-in-water emulsions with the capacity to disperse the oil in the liquid phase. This behavior is not observed by mono-RM due to its greater hydrophobicity, which makes this surfactant stay more at the solid–liquid interface. Moreover, it is important to emphasize that RMLs used consist of the culture media supernatant freeze-dried with a purity of around 10–15%, therefore, other components of the media could also interfere in the process.

This aligns with previous studies, such as Fang et al.^6^ who evaluated the use of different surfactant mixtures with HLB ranging from 4.3 to 15 in the DCO recovery from condensed corn distillers’ solubles. The HLB of the different surfactant mixtures has a significant impact on oil recovery, with an increase in oil recovery with the increase in HLB from 4.3 to 9.7 and a decrease in oil recovery with an HLB higher than 9.7. Therefore, the mixture with an HLB of 9.7 promoted the highest oil recovery.

The addition of Tween 80 in ethanol fermentation media was previously reported by Fang et al.^5^ The authors evaluated the addition of Tween 80 concentrations ranging from 0.2 to 1 g/L and found that there was a significantly higher DCO migration to the thin vinasse with the addition of 0.5 g/L Tween 80, and further increases in surfactant concentration did not improve the oil migration, probably due to the oil enclosed in the unbroken cells. This higher migration was attributed to the removal of the oil adhered to solid particles from the aqueous phase.

The higher oil recovery from thin vinasse corroborates with the visual aspect of the bioreactor after ethanol production with di-RML, where a clear separation of oil in the vinasse could be noticed, an aspect that is not observed in ethanol production without a surfactant (Figure 4). The biosurfactants efficiently separated distiller’s corn oil (DCO) from the fermentation media, demonstrating their potential in improving oil recovery. This efficiency can be attributed to the demulsifying action of biosurfactants, which remove oil adhered to corn bran and emulsify with proteins.

**Figure 4:**
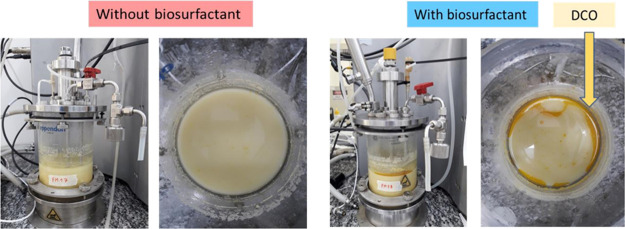
Fermentation media after ethanol evaporation and centrifugation. (A) Control, without surfactant addition. (B) With biosurfactant addition, in this case, a visual separation of the distiller’s corn oil (DCO) was noticed at the top.

Moreover, it is important to highlight that the surfactant addition did not impact the ethanol productivity. In all conditions, the ethanol concentration was around 80 g/L after 24 h, providing an ethanol yield (Yp/s) of Yp/s = 0.26 ± 0.01 and volumetric productivity (Qp) of 3.29 ± 0.12 g/(L h). This is crucial from an industrial perspective as it demonstrates that these biosurfactant molecules can be incorporated into corn ethanol production without altering its productivity.

These findings align with previous studies, such as by Fang et al.,^5^ who reported no negative interference of surfactants on ethanol production. Instead, surfactants were found to enhance the process by modifying the crystallinity and morphology of corn, thereby reducing the enzyme dosage and improving the ethanol yield.

This study confirms the potential use of biosurfactants in the ethanol production process for oil recovery improvement from fermented solids, providing a new, environmentally friendly option in the biorefinery context. Biosurfactants can serve as a viable alternative to traditional chemical surfactants, which align with the goal of creating more sustainable industrial processes by reducing the reliance on chemical surfactants. Furthermore, the addition of biosurfactants has the advantage of producing a solid residue (that will be used to produce DDGS) with a lower fat content, which is desired, as it enhances the DDGS palatability, thereby improving the quality of this coproduct for animal feed.^5,23,24^ Moreover, this produced DDGS probably has a residue of RML, a potential animal feed additive, since the dietary supplementation of poultry with RML may improve growth performance and immune and antioxidant function and regulate broilers' intestinal microbiota.^25–27^

### 3.3 Impact of Biosurfactants on Viscosity

In addition to the benefits of aiding in DCO recovery, it was evaluated that the addition of surfactants reduced the viscosity compared to the control. This evaluation was performed for the control condition (without addition of a surfactant) and for the condition with di-RML (0.5 g/L) since this biosurfactant effectively aids in a greater recovery of oil in the thin vinasse. Table 2 presents the corn slurry viscosity during the ethanol production steps, gelatinization, liquefaction, and simultaneous saccharification and fermentation, with and without di-RML addition.

**Table 2:** Corn Slurry Viscosity (cSt) over Ethanol Production Stages with Di-RML (0.5 g/L) and without RML (Control Condition)^a^

	gelatinization	liquefaction	simultaneous saccharification and fermentation
control (without RML)	11.5 ± 0.2^b^	11.1 ± 0.6^a,b^	9.98 ± 1.09^a^
di-RML (0.5 g/L)	10.8 ± 0.3^c^	8.17 ± 0.42^a^	9.05 ± 0.29^b^

a Results are expressed as means ± SD for triplicates. Different letters in the same line represent significantly different values (*p* < 0.10).

In the ethanol production without di-RML, the viscosity remains statistically stable between the gelatinization and liquefaction steps; however, the viscosity of the simultaneous saccharification and fermentation step is lower than the gelatinization. However, in ethanol production with di-RML, an expressive viscosity reduction occurs between the gelatinization and liquefaction steps, followed by a slight increase in the simultaneous saccharification and fermentation step.

During ethanol production, the viscosity is expected to change during the steps. In the gelatinization step, starch gelatinizes. During this step, the corn kernels absorbed water (viscosity increases) until they deconstructed (viscosity decreases). This step occurs at 65 °C. Subsequently, the temperature is increased to 80 °C to allow the action of α-amylase, which hydrolyzes the starch into smaller oligomers. After this step, simultaneous saccharification and fermentation occurs, where the glucoamylase enzyme hydrolyzes the starch oligomers into glucose, and the yeast preferentially uses glucose as a carbon source for ethanol production. Therefore, as starch is transformed from a larger polymer into smaller molecules, the viscosity is expected to decrease with process progress. Indeed, in ethanol production, a drop in viscosity has been observed in the simultaneous saccharification and fermentation step.

With the addition of the biosurfactant di-RML, there was a significant reduction of the liquefaction and simultaneous saccharification and fermentation viscosities, possibly due to its ability to reduce surface and interfacial tension between liquid and solid, facilitating mixing and decreasing flow resistance.^28^ This reduction is more pronounced during the liquefaction stage, possibly due to the temperature of this stage (80 °C), which favors greater fluid flow, and because of this, the viscosity is higher at the stage of simultaneous saccharification and fermentation than at the liquefaction stage.

The viscosity reduction is desirable since its high viscosity can complicate solid–liquid separation and fermentation efficiency as well as increase handling challenges and incomplete hydrolysis into fermentable sugars. Although excessive water addition can reduce viscosity, it also lowers the concentration of fermentable sugars and increases the energy requirements for water evaporation. Additives that reduce viscosity, like biosurfactants used in this study, are therefore highly beneficial, promoting higher corn slurry fluidity during the ethanol production process, ensuring less encrustation in the pipes, and consequently improving fermentation efficiency. Moreover, Zhang et al.^29^ and Srichuwong et al.^30^ linked viscosity reduction to energy savings; therefore, it is expected that the decrease in viscosity of the corn slurry, promoted by the addition of di-RML, will positively impact the power consumed by the bioreactor engine, thus saving energy from ethanol plants.

## 4 Conclusions

This study demonstrates the efficacy of rhamnolipid biosurfactants in enhancing the recovery of distiller’s corn oil (DCO) during ethanol production for the first time. The three surfactants evaluated (mono-RML, di-RML, and Tween 80) allowed a total oil recovery (from solids and thin vinasse) of nearly 90–95%, where at the condition without surfactants, only around 70% was achieved. Moreover, regarding the oil extraction from thin vinasse, the addition of di-RML and Tween 80 in the ethanol production process allowed a significantly higher oil recovery in this fraction when compared to the control. The results indicate that di-RML performs comparably to the industrial chemical surfactant Tween 80 in oil recovery without negatively impacting ethanol production. This higher oil recovery benefits producing animal feed since it allows the production of a residual solid with a lower fat content and, consequently, better palatability. The addition of di-RML also significantly reduced the viscosity of the corn slurry during liquefaction and simultaneous saccharification and fermentation steps compared to that of the control. This reduction in viscosity translates to lower energy costs and decreased fouling in the evaporators of corn ethanol plants. Furthermore, the biosurfactants efficiently demulsified commercial DCO, removing residual proteins from the fermentation process, which can be used for other applications, such as animal feed supplementation. Moreover, it is also worth mentioning that the RMLs used in this study used DCO as a carbon source for its production, making this additive even more interesting in a biorefinery context, where byproducts are used to increase the effectiveness and sustainability of the process itself. Therefore, the addition of di-RML as an additive in ethanol production proved to be efficient in aiding in greater recovery of DCO; however, tests related to the optimization of the concentration of di-RML used and the impact on process costs still need to be evaluated.
